# Prevalence and predictors of glucose metabolism disorders among People Living with HIV on combination antiretroviral therapy

**DOI:** 10.1371/journal.pone.0262604

**Published:** 2022-01-19

**Authors:** Wondmagegn Tamiru Tadesse, Birhanemeskel T. Adankie, Workineh Shibeshi, Wondwossen Amogne, Eleni Aklillu, Ephrem Engidawork

**Affiliations:** 1 Department of Pharmacology and Clinical Pharmacology, School of Pharmacy, College of Health Sciences, Addis Ababa University, Addis Ababa, Ethiopia; 2 Department of Medical Microbiology, School of Medicine, St. Paul Specialized Hospital Millennium Medical College, Addis Ababa, Ethiopia; 3 Department of Internal Medicine, School of Medicine, College of Health Sciences, Addis Ababa University, Addis Ababa, Ethiopia; 4 Department of Laboratory Medicine, Division of Clinical Pharmacology, Karolinska Institute, Stockholm, Sweden; Purdue University, UNITED STATES

## Abstract

**Objective:**

We investigated prevalence and predictors of glucose metabolism disorders (GMDs) among People Living with HIV (PLWH) on efavirenz- and atazanavir/ritonavir-based combination antiretroviral therapy (cART).

**Methods:**

This cross-sectional study involved adult PLWH on efavirenz- (n = 240) and atazanavir/ritonavir-based (n = 111) cART. The prevalence of GMDs was determined by fasting serum glucose, insulin, and homeostasis model assessment. A logistic regression model was used to determine predictors.

**Results:**

The overall prevalence of GMDs for all regimens was 27.6% (97/351) [95% CI 23.0–32.6%] s, with 31.1% (75/240) [95% CI 25.4–37.5%] for efavirenz-based and 19.8% (22/111) [95% CI 12.9–28.5%)] for atazanavir/ritonavir-based cART group. The prevalence of impaired fasting glycemia was significantly higher (p = 0.026) in the efavirenz- [(15.4%) (37/240); 95%CI (11.1–20.6%)] than atazanavir/ritonavir-based [(7.2%) (8/111), (95%CI (3.2–13.7%)] cART. However, no significant difference was observed in the prevalence of diabetes mellitus and insulin resistance between the two regimens. Age ≥46 years old and specific type of ARV contained in cART, such as TDF, were independent predictors of GMD in both groups. Whereas the male gender and BMI category were predictors of GMDs among EFV-based cART group, AZT- and ABC- containing regimens and triglyceride levels were predictors in the ATV/r-based group.

**Conclusions:**

GMDs were highly prevalent among adults on EFV- than ATV/r-based cARTs. Age ≥46 years and TDF-containing cARTs are common predictors in both regimens. Close monitoring for impaired fasting glucose during long-term EFV-based cART is recommended for early diagnosis of type-2 diabetes and management.

## Introduction

HIV/AIDS has remained a public health problem in sub-Saharan Africa [1]. Over the last three decades, HIV-associated mortality and disease transmission rate has progressively declined, mainly because of the rapid expansion and availability of combination Antiretroviral Therapy (cART) [2]. The introduction of cART has changed the complexion of HIV infection from a deadly disease to a chronic manageable disorder that notably changed patients’ quality of life and longevity [3]. It also changed the global epidemiology of transmission, morbidity, and mortality of HIV [2, 4].

Treatment of HIV is lifelong, embracing frequent clinical evaluation and follow-up [5]. During long-term exposure to antiretroviral therapy (ART), individual patients may experience treatment-associated adverse events or drug toxicities [5]. Long-term exposure to ART may increase the risk of metabolic abnormalities such as lactic acidosis, osteopenia, dyslipidemia, and glucose metabolism disorders (GMDs) [6]. GMDs are glucose homeostasis dysregulations that include diabetes mellitus (DM), impaired glucose tolerance (IGT), impaired fasting glycemia (IFG), or insulin resistance (IR) [7].

The literature indicates that the prevalence of IR, IGT, and DM has significantly increased and became a notable clinical concern, as long-term ART [8] and aging-related factors contribute to a higher risk of glucose metabolism abnormalities. DM is now emerging as one of the non-infectious comorbid conditions among People Living with HIV (PLWH) on cART [8]. Previous studies reported that patients on ART were found to be four-fold more prone to DM and associated conditions compared to HIV uninfected individuals [6, 7, 9–11]. Among cART classes, protease inhibitors (PI) use has been commonly reported to have association with GMDs. PIs may cause abnormal glucose metabolism, ranging from IR through IGT & IFG to type 2 diabetes [8, 12]. The PIs, particularly lopinavir and ritonavir, are linked to an increase in IR and to have effect on lipid and glucose metabolism [13, 14]. A cross-sectional study reported a five-to-nine-fold elevated prevalence of type 2 DM among PLWH on PIs [12]. Recently, a high prevalence of cART-associated dyslipidemia, particularly low High-Density Lipoprotein Cholesterol (HDL-c) and hypertriglyceridemia, has also been reported among treatment-experienced HIV-infected children from Ethiopia [15].

In a resource-limited setting, Efavirenz (EFV) and Atazanavir/Ritonavir (ATV/r) serve as a backbone of combination antiretroviral regimens [16]. In Ethiopia, during the study period, the preferred first-line regimen for adults was EFV-based cART, specifically a combination of tenofovir (TDF), lamivudine (3TC), and EFV [17]. ATV/r-based regimens were the mainstay of second-line cART replacing lopinavir/ritonavir (LPV/r) [17].

The magnitude of and risk factors for GMDs are well investigated in developed countries. Several studies from sub-Saharan African countries also reported a high prevalence of glucose-related abnormalities and risk factors. However, regimen-specific prevalence and predicting factors, particularly for EFV- and ATV/r-based regimens, are limited in Ethiopia. Principally, data are almost unavailable concerning glucose metabolism-related alterations of ATV/r-based regimens, at least in Ethiopia. Moreover, data comparing the incidence of GMDs and the respective risk factors of Non-Nucleoside Reverse Transcriptase Inhibitors (NNRTIs), including EFV, and new PIs, such as atazanavir (ATV) are sparse. Thus, this study would provide data and generate evidence for interested researchers and clinicians in sub-Saharan Africa in general and in Ethiopia in particular. Therefore, this study aimed to determine the prevalence and predicting factors of GMDs among PLWH on EFV- and ATV/ritonavir (ATV/r)-based cARTs.

## Methods

### Study design, population, and setting

This is an institution-based cross-sectional study conducted among treatment-experienced PLWH on EFV- or ATV/r-based cART. The study was conducted from August 2019 to March 2020 at the ART clinic of Tikur Anbessa Specialized Hospital (TASH), College of Health Sciences, Addis Ababa University, Addis Ababa, Ethiopia. TASH is the largest tertiary level teaching and referral hospital in Ethiopia. This setting regularly receives referred patients from different parts of the country. With more than 800 beds, it provides various specialized clinical services for more than 500,000 patients each year [18]. The ART clinic at TASH provides HIV/AIDS prevention, patient care, and ART services.

All adult PLWH on EFV- or ATV/r- based ART attending at ART-clinic of TASH formed the study population. Study participants were confirmed PLWH recruited based on the inclusion and exclusion criteria.

### Sample size determination and sampling techniques

The sample size was determined using single proportion formula for cross-sectional studies, with a qualitative variable [19]. To estimate the specific prevalence for sample size calculation, the sum of the prevalence of IFG (pre-diabetes) and DM in PLWH on ART were considered from two local cross-sectional studies [20, 21]. Accordingly, a prevalence of 31.2% was calculated. In addition, 90% power to detect a prevalence difference of 10% between the two groups, 95% confidence interval, and 0.05 level of significance was considered for sample size calculation using the single proportion formula.

The calculated sample size needed for the cross-sectional study was therefore about 330 patients. Adding a 10% contingency for the probability of missing data, the total sample size reached to 363.

The overall proportion of patients receiving EFV- and ATV/r-based cART during the study period was 69% and 31%, respectively. Accordingly, 251 participants were recruited from those on EFV-based cART and 112 from those on ATV/r-based cART. A convenient sampling technique was used to recruit study participants based on their consent and inclusion/exclusion criteria.

PLWH aged 18 years and above and on EFV or ATV/r-based cART at least for one year were included. Patients known to have DM, pregnancy, cancer, renal disease, liver disease, uncontrolled hypertension, and heart failure were excluded from the study. Moreover, patients on certain co-administered medications such as antipsychotics, cancer chemotherapy, anti-TB, corticosteroids, hormonal agents, or antidiabetics were excluded.

### Data collection

Relevant data including socio-demographic, clinical characteristics, adherence based on self-reported 3 days recall test, in which study participants were asked to report the number of doses they missed over the last three consecutive days prior to sampling date [22–24], and anthropometric measurement were collected using a semi-structured interview questionnaire, patient medical charts, and prospective laboratory sample analysis. Waist circumference and body weight were measured with participants wearing light clothing and barefooted. Waist circumference was measured at the umbilical level to the nearest 0.1 cm using a tape measure. BMI was computed as weight divided by height square (kg/m^2^). Waist circumference and BMI were defined according to WHO recommendations [25].

Blood tests were performed after overnight fasting (8 to 12 h). About 5 ml blood was collected from the brachial artery in serum separator tubes and fasting blood glucose (FBG), insulin, and lipid profiles were determined.

### Operational definitions

- DM was defined as a fasting glucose level of 126 mg/dL or higher [26].
- IFG was defined as a fasting glucose level between 110 and 125 mg/dL [26].
- Normoglycemia was defined as a fasting serum glucose level between 70 and 109 mg/dL [26, 27].
- Hyperglycemia was defined as a fasting glucose level of 110 mg/dL or higher [26, 27].
- IR was diagnosed by either Homeostasis model assessment insulin resistance (HOMA-IR) value of ≥3.8, fasting plasma insulin of ≥20 μU/ml, or fasting glucose/insulin ratio of ≥4.5 [28, 29].
- GMDs were defined as the presence of IFG, IR, or DM [7, 30].

### Data management and analysis

Data were sorted and entered as codes suitable for Statistical Package for Social Science (SPSS) statistical software version 25. Socio-demographic, anthropometric, and clinical as well as laboratory results were presented using descriptive statistics (frequency, mean, median, interquartile range). Continuous variables were reported as mean ± standard error of the mean (SEM), while categorical variables were presented as percent proportions. HOMA-IR was calculated to determine IR using FBG level and insulin concentrations. HOMA-IR is given by the product of fasting insulin concentration (μU/ml) and fasting glucose (mmol/L) level divided by the constant normalizing factor, 22.5 [31].

To determine associations of variables against GMDs, univariate logistic regression analysis was performed for each socio-demographic, anthropometric, and clinical lab variables. Multivariate analysis was performed using backward-stepwise logistic regression analysis and independent predictors of the primary outcome were identified. Variables with a p-value less than 0.05 were considered statistically significant, while variables with p values less than 0.2 in univariate analysis were candidates for multivariate logistic regression analysis.

### Ethical consideration

Ethical clearance was obtained from the Institutional Review Board of College of Health Sciences, Addis Ababa University (Protocol No. 019/19/SoP) and National Ethical Review Committee, Ministry of Science and Higher Education, Addis Ababa, Ethiopia (Ref. No. MoSHE/RD14.1/9324/20). Written informed consent was obtained from each study participant after a full explanation of the purpose and nature of all procedures used. In cases of severe abnormal values, participants were contacted through the HIV clinic and referred for a timely clinical evaluation and follow-up at the same hospital.

## Results

### Baseline characteristics of study participants

Out of the 363 recruited study participants, 351 had complete clinical laboratory data for FBG fasting serum insulin, and HOMA-IR value, and thus considered for statistical analysis. As depicted in Table 1, there was a female preponderance in the study participants (70.4%), EFV-(68.8%), and ATV/r-based (73.9%) regimens. Majority of the participants (58.4%) belong to the age group of 18–45 years, with 54.6% and 66.7% in EFV- and ATV/r-based cART group, respectively. A large proportion of participants were non-smokers (99.1%), non-khat chewers (98.6%), or non-alcohol users (96.6%). Related to anthropometric characteristics, the overall mean (SEM) lean weight was 62.4 (0.71) kg, waist circumference was 34.11 (0.26) cm, and BMI was 24.0 (0.26) kg/m^2^.

**Table 1:** Baseline characteristics of study participants on efavirenz-based or ritonavir-boosted combination antiretrovirals.

Variables	Categories	Overall	EFV-based	ATV/r-based	χ^2^/F or t	p
		n (%)	n (%)	n (%)		
Age (years)	Median (IQR)	43.0 (37.0–50.0)	45.0 (38.0–52.0)	40.0 (33.0–48.0)	3.5	0.000
Age category	18≤45	205(58.4)	131 (54.6)	74 (66.7)	4.6	0.033
	≥46 years	146 (41.6)	109 (45.4)	37 (33.3)		
Gender	Female	247 (70.4)	165 (68.8)	82 (73.9)	1.0	0.328
	Male	104 (29.6)	75 (31.3)	29 (26.1)		
Marital status	Single	76 (21.7)	47 (19.6)	29 (26.1)	3.0	0.387
	Married	127 (36.1)	93 (38.8)	34 (30.6)		
	Widowed	86 (24.5)	59 (24.6)	27 (24.3)		
	Divorced	62 (17.7)	41 (17.1)	21 (18.9)		
Educational status	Up to primary	145 (41.3)	96 (40.0)	49 (44.1)	0.9	0.332
	Above primary	194 (55.3)	138 (57.5)	56 (50.5)		
Khat use (self-report)	Never	346 (98.6)	236 (98.3)	110 (99.1)	0.317	0.573
	Current or previous	5 (1.4)	4 (1.7)	1 (0.9)		
Smoking (self-report)	Never	348 (99.1)	238 (99.2)	110 (99.1)	0.004	0.949
	Current or previous	3 (0.9)	2 (0.8)	1 (0.9)		
Alcohol use (Self-report)	Never	339 (96.6)	231 (96.3)	108 (97.3)	0.252	0.616
	Current or previous	12 (3.4)	9 (3.8)	3 (2.7)		
Treatment adherence (3-day test)	Adhered	339 (96.6)	231 (96.3)	108 (97.3)	0.252	0.616
	Non-adhered	12 (3.4)	9 (3.8)	3 (2.7)		
BMI category	<18.5	40 (11.4)	25 (10.4)	15 (13.5)	5.4	0.143
	18.6–24.9	163 (46.4)	105 (43.8)	58 (52.3)		
	25–29.9	104 (29.6)	80 (33.3)	24 (21.6)		
	≥30	34 (9.7)	24 (10.0)	10 (9.0)		
Viral load status(n = 335)	≥1000 copies/ml	31 (8.8)	3 (1.3)	28 (25.2)	61.7	0.000
	<1000 copies/ml	304 (86.6)	225 (93.8)	79 (71.2)		
History of comorbidity (self-reported)	HIV-only	340 (96.9)	233 (97.1)	107 (96.4)	0.118	0.731
	HIV + comorbidity	11 (3.1)	7 (2.9)	4 (3.6)		
cART backbone-type		351 (100)	240 (68.4)	111 (31.6)		
Specific ARVs contained in cART	TDF containing	266 (75.8)	216 (90.0)	50 (45.0)	80.9	0.000
	AZT containing	71 (20.2)	24 (10.0)	47 (42.3)	45.3	0.000
	ABC containing	13 (3.7)	-	13 (11.7)	29.2	0.000
Time since HIV confirmed date (months) ^δ^		131.14±2.6	134.5±3.1	123.9±4.9	1.9	0.062
Cumulative time on cART (month) ^δ^		120.2±2.4	123.9±2.9	112.0±4.3	2.3	0.021
Cumulative time on EFV-based 1^st^-line (month) ^δ^		82.3±2.7	101.4±2.8	40.8±4.2^#^	12.2	0.000
Cumulative time on ATV/r-based 2^nd^-line (month) ^δ^		11.3±1.3	0.7±0.5*	34.3±3.0	115.1	0.000
Time on current cART regimen type (month) ^δ^		74.6±2.5	93.7±2.7	33.1±2.6	16.2	0.000
Time on prior cART regimen types (month) ^δ^		52.4±5.6	34.8±5.6	90.5±11.9^#^	349	0.000
Time on NVP-based 1^st^-line (Prior to EFV) (month) ^δ^		25.4±2.1	21.7±2.4	33.4±4.3^#^	178.7	0.018
Time on LPV/r-based 2^nd^-line (prior to ATV/r) (month) ^δ^		1.4±0.5	0.2±0.2*	4.1±1.6	112.6	0.018
Waist circumference (cm) ^δ^		34.1±0.26	34.6±0.3	33.3±0.4	2.4	0.016
CD4+ (cells/ul) (baseline) ^δ^		206.0±18.3	225.5±25.6	163.3±16.2	1.6	0.114
CD4+ (cells/ul) (recent) ^δ^		464.6±13.6	523.5±16.4	336.8±19.3	7.4	0.000
Fasting glucose (mg/dL) ^δ^		99.2±1.5	101.7±1.7	94.2±2.6	2.4	0.018
Fasting insulin (uU/ml) ^δ^		9.6±0.6	10.2±0.8	8.4±0.6	1.4	0.150
HOMA-IR (μU/ml) ^δ^		2.5±0.2	2.8±0.3	2.0±0.2	1.4	0.154
Total cholesterol(mg/dL) ^δ^		200.8±6.9	208.5±9.9	184.2±4.1	1.6	0.103
Triglyceride(mg/dL) ^δ^		155.9±4.0	152.2±5.0	163.7±6.4	1.3	0.179
HDL-C(mg/dL) ^δ^		45.3±1.7	46.3±1.8	43.1±3.6	0.9	0.370
LDL-C(mg/dL) ^δ^		126.1±6.3	135.6±9.0	105.5±3.4	2.2	0.026

Values are frequencies or ^δ^mean ± SEM (N = 351), TDF = Tenofovir, AZT = Zidovudine, ABC = Abacavir, HDL-C = High density lipoprotein Cholesterol, LDL-C = Low density Lipoprotein Cholesterol.

^#^Prior regimens in subsequent order of NVP-based, EFV-based, and LPV/r-based before switched to the current ATV/r-based 2^nd^-line cART.

*Few study participants had a switch to 2^nd^-line between initiation and current 1^st^-line cART. HIV Comorbidities refer to conditions such as dyslipidemia, hepatitis or asthma based on patients’ report. χ^2^ = chi-square test for categoric variables, F = F-test for categoric variables with 1 cell expected count <5 or t = independent t-test for continuous variables.

About 240 (68.4%) of the participants were on EFV-based 1^st^ line cART (Table 1). The overall mean (±SEM) of baseline and latest CD4 counts were 206 ±18.3 and 464.6 ±13.6, respectively. Unlike the baseline (225.5±25.6 vs. 163.3±16.2), a significantly elevated latest (523.5±16.4 vs. 336.8±19.3) CD4 counts were observed in the EFV- than ATV/r-group. Based on the latest medical records, only 31 (8.8%) of the overall study participants experienced virologic failure, with viral loads of >1000 copies/ml, out of which a significant majority were on ATV/r-based cART (p = 0.000). The EFV group had a significantly longer cumulative time on cART since initiation (123.9±2.9 months) than the ATV/r-group (112.0±4.3 months) (Table 1). Whilst the cumulative time on EFV-based regimen was 101.4±2.8, it was 34.3±3.0 months for ATV/r-based cART. Participants on ATV/r-based cART were on 1^st^-line cART for 90.5±11.9 months, mainly on Nevirapine (NVP)-based for 33.4±4.3 months and later, on EFV-based regimens for 40.8±4.2 months.

Large majority of the participants (339, 96.6%) were adherent to their respective cART based on the self-reported three-day adherence test. Treatment adherence was higher among participants in ATV/r- (97.3%) than EFV-based groups (96.3%), though no statistically significant difference was found (p = 0.616). Based on the overall clinical lab analysis, the mean (SEM) FBG was 99.2 (1.5) mg/dL and serum insulin ranged from 0.46 to 160.5 μU/mL, with a mean (SEM) of 9.6 (0.6) (Table 1). Moreover, the mean (SEM) HOMA-IR value was found to be 2.5 (0.2), ranging from 0.14 to 57.39. Unlike fasting serum insulin and HOMA-IR values, the EFV-based group showed a significantly elevated FBG than ATV/r-based (p = 0.018). In general, the clinical lab values were relatively elevated in the EFV- than the ATV/r-based group, except for the triglyceride level. However, only LDL level showed a statistically significant elevation among EFV- than ATV/r-based cART receiving group (Table 1).

### Prevalence of GMDs

The prevalence of GMDs is shown in Table 2. The overall prevalence of GMDs was found to be 27.6% (n = 97/351). Among the overall study participants, about 12.8% (45/351) were with impaired glycemia and 5.7% (20/351) with a diabetic range of fasting serum glucose. IR was detected in about 14.8% (n = 52/351) of the study participants. A significantly higher (p<0.05) prevalence of GMDs was found in patients taking EFV-based first-line therapy (31.3%) than ATV/r therapy (19.8%). Disaggregating the data revealed that only the prevalence of IFG was found to be significantly higher (p<0.05) in the EFV- (15.4%) than the ATV/r-based group (7.2%). Although the prevalence of DM and IR tended to be higher in the EFV group than the ATV/r group, it did not reach statistical significance (Table 2).

**Table 2:** Prevalence of glucose metabolism disorders relative to the overall and specific type of combination antiretroviral treatment category among the study participants.

Variables	Overall (N = 351)	EFV-Based cART (n = 240)	ATV/r-Based cART (n = 111)	χ^2^	p
Impaired Fasting Glycemia	45 (12.8%)	37 (15.4%)	8 (7.2%)	5.0	0.026
Diabetes Mellitus	20 (5.7%)	17 (7.1%)	3 (2.7%)	1.5	0.318
Insulin Resistance	52 (14.8%)	36 (15%)	16 (14.4%)	0.1	0.748
Glucose Metabolism Disorders	97 (27.6%)	75 (31.3%)	22 (19.8%)	4.6	0.039

We also tried to calculate the prevalence of these variables based on tenofovir (TDF)-, zidovudine (AZT)-, and abacavir (ABC)-containing combinations. The prevalence of GMDs was higher in participants taking AZT- (29.6%) than TDF- (27.3%) and ABC (15.4%)- containing combinations. Looking at the individual variables, the AZT-containing combination showed a relatively higher rate for IR (18.3%) and IFG (14%) than TDF-(13.9% for IR and 13.1% for IFG) and ABC- (7.7% for IR and 0% for IFG) containing combinations. By contrast, prevalence of DM was higher for ABC- (7.7%) than TDF- (5.6%), and AZT- (2.8%)-containing combinations.

### Predictors of glucose metabolism disorders

#### Predictors for overall study participants

Univariate analysis revealed that BMI, serum level of triglycerides, age, gender, khat use, comorbid conditions, and history of hypertension were significantly associated with GMDs (Table 3). However, in multivariate logistic regression analysis, age ≥46 years old, male gender, history of comorbid conditions, and serum triglycerides level were found to be independent predictors of GMDs.

**Table 3:** Predictors of glucose metabolism disorders determined by logistic regression analysis among all study participants (n-97).

Variables	Categories	Univariate		Multivariate	
		COR	P	AOR	P
Age category	≤45	1	-	1	0.006
	≥46	2.3 (1.4, 3.8)	0.000	2.1(1.2, 3.6)	
Gender	Female	1		1	0.001
	Male	3.2 (2.0, 5.3)	0.000	2.6 (1.4, 4.5)	
Educational status	Up to Primary	1	-		
	Above primary	1.6 (0.9, 2.5)	0.063		
Marital status	Single	1			
	Married	1.0 (0.6,2.0)	0.913		
	Widowed	0.9 (0.4,1.8)	0.768		
	Divorce	1.1 (0.5, 2.3)	0.856		
Ever khat use (self-report)	Never	1	0.034		
	Current or previous	10.9 (1.2, 98.6)			
Ever smoking (self-report)	Never	1	-		
	Current or previous	5.3 (0.5, 59.4)	0.174		
Ever alcohol use (Self-report)	Never	1	-		
	Current or previous	1.9 (0.6, 6.2)	0.276		
Treatment adherence (3-day test)	Non-adhered	1	-		
	Adhered	4.3 (0.5, 34.1)	0.162		
BMI category	≥30	1			
	<18.5	0.13 (0.04, 0.43)	0.001		
	18.6–24.9	0.5 (0.2, 1.0)	0.066		
	25–29.9	0.4 (0.2, 1.1)	0.074		
CD4 (n = 342) (recent)	<350	1			
	≥350	0.9 (0.6, 1.5)	0.762		
Viral load status(n = 335)	≥1000 copies/ml	1	-		
	<1000 copies/ml	1.3 (0.6, 3.2)	0.524		
History of comorbidity (self-reported)	HIV-only	1	-	1	0.027
	HIV + comorbidity	4.9 (1.4, 17.0)	0.013	4.7 (1.2, 18.4)	
cART backbone-type	ATV/r-based	1	-		
	EFV-Based	1.8 (1.1, 3.1)	0.027		
Specific ARVs contained in cART	TDF containing	0.9 (0.5, 1.6)	0.826		
	AZT containing	1.1 (0.6, 2.0)	0.682		
	ABC containing	0.5 (0.1, 2.1)	0.325		
Time since HIV confirmed date (months)		1.003(0.998, 1.008)	0.243		
Cumulative time on cART (month) ^δ^		0.993 (0.992, 0.995)	0.0.00		
Cumulative time on EFV-based 1^st^-line (month) ^δ^		0.993 (0.990, 0.995)	0.000		
Cumulative time on ATV/r-based 2^nd^-line (month) ^δ^		0.966(0.952,0.980)	0.000	0.98(0.96,0.99)	0.011
Time on current cART regimen type (month) ^δ^		0.992(0.989,0.995)	0.000		
Time on prior cART regimen types (month) ^δ^		0.989(0.985,0.993)	0.000		
Time on NVP-based 1^st^-line (Prior to EFV) (month) ^δ^		0.989(0.984,0.995)	0.000		
Time on LPV/r-based 2^nd^-line (prior to ATV/r) (month) ^δ^		1.0(0.98,1.02)	0.907		
Waist circumference (cm)		1.1 (0.99, 1.11)	0.055	0.96(0.94,0.99)	0.002
Total cholesterol (mg/dL)		1 (0.998, 1.002)	0.972		
Triglyceride (mg/dL)		1.008 (1.004, 1.011)	0.000	1.005 (1.001, 1.008)	0.017
HDL-C (mg/dL)		0.997 (0.987, 1.007)	0.566		
LDL-C (mg/dL)		1.001 (0.999, 1.002)	0.587		

Patients with age ≥46 years [AOR = 2.1, 95% CI 1.2–3.6, p<0.01] and males had a two-fold risk of GMDs [AOR = 2.6, 95% CI 1.1–3.5, p<0.01]. Likewise, individuals with comorbid conditions had a nearly five-fold risk of GMDs [AOR = 4.7, 95% CI 1.3–18.9, p<0.05] than those without comorbid conditions. Serum level of triglycerides showed a statistically significant association with the incidence of GMDs. For each unit increase in the level of triglycerides, the likelihood to develop GMDs increased by 0.5% [AOR = 1.005, 95% CI 1.001–1.008), p<0.05].

On the other hand, cumulative time on ATV/r-based second-line cART conferred a significant protection from GMDs, *i*.*e*., a 2% lower incidence of GMD was observed for each month stay on ATV/r-based cART (Table 3). Paradoxically, waist circumference was negatively associated with the incidence of GMD. A 4% lower incidence of GMD was noted with a 1 cm increase in waist circumference.

#### Predictors for EFV-based cARTs

Being age ≥46 years old, male gender, BMI category, and TDF-containing combinations were independent predictors to GMDs (Table 4). A 2.1-fold high risk of GMDs was observed among participants with age ≥46 years old (AOR = 2.1, 95% CI 1.1–4.0, p = 0.02). The male gender had a 4.3 times higher risk of GMDs (AOR = 4.3, 95% CI 2.2–83, p = 0.000). Underweights (BMI <18.5) had an 80% lower incidence of GMDs (AOR = 0.2, 95% CI 0.04, 0.7, p = 0.014) than obese participants (BMI ≥30). Likewise, TDF consisting regimens demonstrated a lower incidence of GMDs by about 70% (AOR = 0.3, 95% CI 0.1, 0.6, p = 0.001) than AZT consisting EFV-based cARTs. This was consistent with chi-square analysis, which found a significant association of GMDs with AZT-containing EFV-based regimen than AZT-containing ATV/r-based cART (χ^2^ = 6.3, p = 0.012).

**Table 4:** Predictors of glucose metabolism disorders among EFV- (n = 75) and ATV/r-based (n = 22) cART receiving groups.

Variables		Categories	EFV-based				ATV/r-based			
			Univariate		Multivariate		Univariate		Multivariate	
			COR	p	AOR	p	COR	p	AOR	p
Age category		≤45	1		1		1		5.6(1.6,19.3)	0.006
		≥46	0.5 (0.3,0.8)	0.006	2.1(1.1, 4.0)	0.02	0.4 (0.2, 1.1)	0.069		
Gender		Female	1		1		1			
		Male	4.6 (2.6,8.4)	0.000	4.3 (2.2, 8.3)	0.000	1.1 (0.4,3.1)	0.891		
Educational status		Up to primary	1				1			
		Above Primary	1.8 (0.99,3.1)	0.055			1.1 (0.4, 2.9)	0.868		
Marital status		Single	1				1			
		Married	0.5 (0.3,0.8)	0.002			0.2 (0.1,0.4)	0.000		
		Widowed	0.3 (0.2,0.9)	0.000			0.4 (0.1,0.8)	0.017		
		Divorce	0.5 (0.2, 0.9)	0.022			0.3 (0.1,0.9)	0.023		
Treatment adherence (3-day test)		Non-adhered	1				1			
		Adhered	0.48 (0.37,0.63)	0.000			0.24 (0.15,0.39)	0.000		
BMI category		≥30	1				1			
		<18.5	0.1 (0.04, 0.46)	0.001	0.2 (0.04,0.7)	0.014	0.1 (0.01,0.54)	0.011		
		18.6–24.9	0.7 (0.5,0.99)	0.042	0.8 (0.3,1.7)	0.499	0.3 (0.1, 0.5)	0.000		
		25–29.9	0.4 (0.23,0.62)	0.000	0.5 (0.2,1.1)	0.096	0.3 (0.1, 0.7)	0.008		
CD4 (n = 342) (recent)		<350	1				1	0.000		
		≥350	0.4 (0.3, 0.6)	0.000			0.2 (0.1,0.4)			
Viral load status(n = 335)		≥1000 copies/ml	1				1	0.000		
		<1000 copies/ml	0.5 (0.3, 0.6)	0.000			0.2 (0.1,0.4)			
History of comorbidity (self-reported)		HIV-only	1				1	1.000		
		HIV+comorbidity	2.5 (0.5,12.9)	0.273			1.0 (0.1, 7.1)			
Specific ARVs contained in cART	TDF containing	No	1		1	-	1		1	0.03
		Yes	0.4 (0.3,0.6)	0.000	0.3 (0.1,0.6)	0.001	0.2 (0.1,0.5)	0.000	0.101 (0.013,0.802)	
	AZT containing	No	1				1		1	0.016
		Yes	0.9 (0.4,2.0)	0.842			0.2 (0.1,0.5)	0.000	0.092 (0.013,0.637)	
	ABC containing	No					1		1	0.012
		Yes					0.2 (0.04,0.8)	0.027	0.034 (0.002,0.474)	
Time since HIV confirmed date (months)			0.995 (0.993,0.997)	0.000			0.989 (0.986,0.997)	0.000	0.99 (0.98,1.0)	0.054
Cumulative time on cART (month) ^δ^			0.995 (0.993,0.997)	0.000			0.989 (0.985,0.993)	0.000		
Cumulative time on EFV-based 1^st^-line (month) ^δ^			0.994 (0.991,0.996)	0.000			0.984 (0.975,0.992)	0.000		
Cumulative time on ATV/r-based 2^nd^-line (month) ^δ^			1.0(0.97,1.04)	0.891			0.961(0.946,0.977)	0.000		
Time on current cART regimen type (month) ^δ^			0.993(0.991,0.996)	0.000			0.968(0.954,0.982)	0.000		
Time on prior cART regimen types (month) ^δ^			0.992 (0.986, 997)	0.003			0.987(0.981, 0.992)	0.000		
Time on NVP-based 1^st^-line (prior to EFV) (month) ^δ^			0.992(0.986,0.999)	0.017			0.985(0.977,0.994)	0.001		
Time on LPV/r-based 2^nd^-line (prior to ATV/r) (month) ^δ^			1.7	1.000			1.0(0.98,1.02)	0.728		
Waist circumference (cm)			0.979 (0.972,0.987)	0.000			0.961 (0.948,0.975)	0.000		
Total cholesterol (mg/dL)			0.996 (0.995,0.998)	0.000			0.993 (0.99,0.995)	0.000		
Triglyceride (mg/dL)			0.997 (0.996,0.999)	0.001			0.994 (0.991,0.996)	0.000	1.009(1.002,1.016)	0.014
HDL-C (mg/dL)			0.984 (0.978,0.99)	0.000			0.967 (0.955,0.978)	0.000		
LDL-C (mg/dL)			0.996 (0.994,0.998)	0.000			0.988 (0.983,0.992)	0.000		

#### Predictors for ATV/r-based cARTs

Being age ≥46 years old, type of specific ARVs contained in ATV-based cART, and serum triglycerides level were independent predictors of GMDs incidence (Table 4). Participants with age ≥46 years old exhibited a 5.6 times higher risk of GMDs (AOR = 5.6, 95% CI 1.6, 21.3, p = 0.006) than age ≤45 years. A >90% lower incidence of GMDs was recorded for TDF-, AZT-, and ABC- containing than their corresponding non-containing type of ATV/r-based cARTs (Table 4). Concerning triglycerides level, the likelihood to develop GMDs increased by 0.9% (AOR = 1.009, 95% CI 1.002–1.016, p<0.05) for each unit increase.

## Discussion

Our study aimed at determining the prevalence and predictors of GMDs among EFV and ATV/r-based cART receiving patients. The overall prevalence of GMDs among participants on EFV-based cART was relatively high than ATV/r-based cART. Remarkably, it was shown that EFV-based cART was associated with the occurrence of IFG than ATV/r-based regimen. Our study linked TDF-containing cART as an independent predictor of GMDs in both EFV- and ATV/r-based combination regimens. Our study considered IR along with IFG and DM as a measure of glucose abnormalities unlike other similar studies [9, 21, 32–34]. This may have increased the chance of detecting GMDs in our study while addressing the pathological hierarchy of glucose-related abnormalities, in which most ARVs in cART are implicated.

The prevalence of DM among PLWH on EFV-based cART in the present study (5.7%) is even higher than reported by WHO in 2016 (3.8%) and IDF (3.2%) [35, 36] for the general population of Ethiopia. This finding may be suggestive of a higher prevalence of GMDs among PLWH on cART, especially on NNRTIs like EFV. A comparative study by Levitt *et al*. [8] reported a prevalence of dysglycemia (26.0%) among PLWH on first-line ART, which is similar to the overall prevalence of GMDs in the present study (27.6%). A relatively close prevalence of GMDs (32.7%) was also reported in a study conducted in Tanzania among HIV-infected patients on ART, though the specific ARV drugs were not indicated [7]. Our study reported lower IFG (12.8% vs. 24%) but a higher DM prevalence (5.7% vs. 2%) than the South African study [37] among EFV-based cART treated participants. In addition, the DM prevalence in this study was slightly lower than reported from North-east (8.8%) [21] and North-west (8.8%) [9] Ethiopia. But it is concordant with that reported from another North-west Ethiopian (5.1%) [33] and Zambian (5%) [38] studies. On the other hand, a lower prevalence of IR was observed in this study as compared to the 21% prevalence reported in a longitudinal study by Araujo *et al*. [29] and 34.2% by Guillen *et al*. [10].

In general, a varied prevalence has been reported for IR, IFG and DM across the literature. These discrepancies could be due to variations in methodology. For instance, the above-stated studies from Northwest Ethiopia, Northeast Ethiopia, and Zambia considered IFG and DM, while our study included IR parameters in addition to IFG and DM to determine the prevalence and risk factors [9, 21, 33, 38]. The sensitivity of kits or equipment used to determine fasting glucose might also differ, accounting for the observed differences (we used the Cobas 6000 (c501) analyzer machine and glucometer was used by the Zambian study). Study designs employed could also contribute to the differences. Araujo *et al*. [29] determined the prevalence using a prospective cohort study, recruiting participants from different types of cART, unlike ours which used a cross-sectional study design using only EFV- and ATV/r-based regimens.

Several studies suggested that EFV containing cART is linked with elevated blood glucose levels due to mitochondrial toxicity or IR [34, 39, 40]. This notion could explain why a high prevalence of GMDs was observed among EFV- than ATV/r-based cART receiving study participants. In contrast, a cross-sectional study from Tanzania indicated that neither EFV nor other ARVs had an association with GMDs among HIV-infected patients on cART [7]. Nonetheless, several lines of recent evidence implicated NNRTIs in disturbed glucose metabolism. For example, studies reported that increased fasting plasma glucose, insulin levels, and decreased insulin sensitivity were observed in NNRTI-based regimens, particularly with EFV [34, 39, 40].

This is one of the few studies that assessed GMDs among HIV-infected patients on ATV/r-based cART, particularly in Ethiopia. As ATV/r-based therapy is relatively new to most resource-limited health settings, our study may provide baseline evidence concerning GMDs during ATV/r-based regimen use. Thus, the findings could help guide ARV drug selection when switching to PI-based second line is considered, particularly for the high-risk group of HIV patients. Based on the finding of this study, those participants on ATV/r-based therapies had a low prevalence of GMDs (3.6%), which is similar to the DM estimates of the Ethiopian population for 2016 (3.8%) [35].

The findings highlighted that the type of specific ARVs contained in cART could influence the occurrence of GMDs, as participants on TDF-containing regimens had a significantly reduced risk for developing GMDs than those on without TDF. Considering IR as a component of GMDs, our study is consistent with the interpretation of “The Women’s interagency HIV study” that reported a lack of clear elevation or precise association between cumulative exposure to five NRTIs, including AZT, ABC or TDF, and HOMA [11].

Consistent with previous studies, our study demonstrated that patients above 45 years of age are at higher odds of developing GMDs in both regimen types as well as in the overall study participants. Aging is a well-recognized traditional risk factor for IFG, diabetes or GMDs in general. A rise in the incidence of GMDs might occur among HIV-infected patients because of increased survival or aging in the face of long-term exposure to cART [7, 21, 41]. Our findings also indicated male patients, specifically among EFV-based cART, were at a higher risk of encountering GMDs than females, which is in line with other studies [34, 38, 41–44]. A meta-analysis also found a significantly higher prevalence of IFG in men among the general population of Eastern, Middle, and Southern African countries [45]. It is thus plausible to assume that the same trend might occur in HIV-infected patients on cART. Although direct evidence is lacking, previous studies suggest that in addition to socio-cultural, lifestyle, or behavioral factors; anthropometric, metabolic, and endocrine differences could contribute to the gender disparity in the prevalence of GMDs [46, 47]. It is also suggested that differences in type and composition of fat might have a role in the risk for IFG, IR, or DM between men and women [48].

### Study limitations

The use of a one-time sampling to define glycemic status rather than confirmation on a subsequent day as recommended can be considered as one of the limitations. However, determination of fasting glucose was run three times for each sample and the average was reported, which could probably offset this limitation. Despite the exclusion of participants with DM, our study design lacks excluding study participants who may have other GMDs. Moreover, our study lacks assessing casual association between long term-cART and GMDs as the study did not have control groups and prior baseline data. The study may also share the limitations emanating from the study design effect, as we used a single institution, a cross-sectional study, and a consecutive sampling during recruitment. Hence, the findings might not be extrapolated to the general PLWH receiving treatment in Ethiopia. Nevertheless, one should note that the study site is the largest referral hospital in Ethiopia, where patients from different parts of the country are referred to receive care. Despite the limitations, this study generated findings related to higher prevalence of GMDs among HIV-infected adult patients, particularly those on EFV-based cART. However, future studies with large sample size, comparative case-control, and prospective study design comprising these regimens should be conducted to confirm the predictors and determine if there exist casual relationships between GMDs and long-term cART.

## Conclusions

In conclusion, we report a high prevalence of GMDs, such as IR, IFG, and DM, among PLWH on EFV-based cART. Age 46 and above and TDF-containing cART were common predictors of GMDs in both EFV- and ATV/r-based treatment groups. The male gender and BMI are predictors of GMDs in EFV-based cART group. AZT- containing and ABC-containing ATV/r-based cARTs as well as elevated serum triglycerides are predictors of GMDs in ATV/r-based cART receiving group. Close monitoring for impaired fasting glucose during long-term efavirenz-based cART is recommended for early diagnosis of type-2 diabetes and management.

## Supporting information

S1 Raw data (SAV)
